# Investigating the Effects of Repetitive Paired-Pulse Transcranial Magnetic Stimulation on Visuomotor Training Using TMS-EEG

**DOI:** 10.1007/s10548-024-01071-1

**Published:** 2024-07-27

**Authors:** Ryoki Sasaki, Brodie J. Hand, Wei-Yeh Liao, John G. Semmler, George M. Opie

**Affiliations:** Discipline of Physiology, The University of Adelaide, Adelaide, SA 5005 Australia

**Keywords:** Repetitive paired-pulse transcranial magnetic stimulation, Primary motor cortex, Motor-evoked potential, TMS-evoked potential, Visuomotor training

## Abstract

I-wave periodicity repetitive paired-pulse transcranial magnetic stimulation (iTMS) can modify acquisition of a novel motor skill, but the associated neurophysiological effects remain unclear. The current study therefore used combined TMS-electroencephalography (TMS-EEG) to investigate the neurophysiological effects of iTMS on subsequent visuomotor training (VT). Sixteen young adults (26.1 ± 5.1 years) participated in three sessions including real iTMS and VT (iTMS + VT), control iTMS and VT (iTMS_*Control*_ + VT), or iTMS alone. Motor-evoked potentials (MEPs) and TMS-evoked potentials (TEPs) were measured before and after iTMS, and again after VT, to assess neuroplastic changes. Irrespective of the intervention, MEP amplitude was not changed after iTMS or VT. Motor skill was improved compared with baseline, but no differences were found between stimulus conditions. In contrast, the P30 peak was altered by VT when preceded by control iTMS (*P* < 0.05), but this effect was not apparent when VT was preceded by iTMS or following iTMS alone (all *P* > 0.15). In contrast to expectations, iTMS was unable to modulate MEP amplitude or influence motor learning. Despite this, changes in P30 amplitude suggested that motor learning was associated with altered cortical reactivity. Furthermore, this effect was abolished by priming with iTMS, suggesting an influence of priming that failed to impact learning.

**Supplementary Information:**

The online version contains supplementary material available at 10.1007/s10548-024-01071-1.

## Introduction

Learning new motor skills is an essential aspect of daily life that is associated with neuroplastic changes in the brain. These changes are characterized by the modulation of existing neural communication and the formation of new connections (for review, see Dayan and Cohen 2011). This role of neuroplasticity in mediating motor learning means that factors influencing plasticity induction also have the potential to influence the extent of learning. Given the clear benefits of such capabilities in both healthy and pathological populations, an extensive literature aiming to modulate learning by manipulating plasticity has developed (Jung and Ziemann 2009; Fujiyama et al. 2017; Sasaki et al. 2018; Opie et al. 2020). A popular approach within this literature has been to leverage the concept of metaplasticity, wherein the sign and magnitude of a neuroplastic change is determined by previous activity within the targeted synapses (for review, see Ziemann and Siebner 2008). Within this construct, an intervention able to produce a directed change in brain activity is applied before a period of training to ‘prime’ neuroplastic changes associated with training (Jung and Ziemann 2009; Fujiyama et al. 2017; Sasaki et al. 2018; Opie et al. 2020). This may occur via both homeostatic and non-homeostatic mechanisms, wherein training is associated with increased potentiation of cortical activity and/or greater skill acquisition following a period of depressed or potentiated synaptic activity, respectively (for review, see Müller-Dahlhaus and Ziemann 2015).

The utility of this priming approach has been facilitated in humans by the application of different forms of non-invasive brain stimulation (NIBS). These techniques can induce short-term neuroplastic changes in the brain (Nitsche and Paulus 2000; Stefan et al. 2002; Huang and Rothwell 2004; Peinemann et al. 2004) and have been shown to influence skill acquisition in a metaplastic way (Jung and Ziemann 2009; Jelić et al. 2015; Fujiyama et al. 2017; Sasaki et al. 2018; Opie et al. 2020). Much of the literature investigating the influence of priming NIBS on motor learning has applied more conventional stimulation (e.g., theta burst stimulation [TBS], paired-associative stimulation [PAS], transcranial direct current stimulation [tDCS]). However, we recently demonstrated that I-wave periodicity repetitive paired-pulse TMS (iTMS) is also able to facilitate acquisition of a novel motor skill (Hand et al. 2023). This intervention involves pairs of perithreshold TMS pulses that are applied using an interstimulus interval approximating the timing of the excitatory interneurons recruited within primary motor cortex (M1) by TMS (i.e., the indirect [I] waves) and is known to potentiate cortical excitability in an LTP-like manner (Thickbroom et al. 2006). When applied prior to motor learning, iTMS was found to result in greater acquisition of a novel motor skill, consistent with non-homeostatic metaplasticity. While our previous work demonstrates the utility of iTMS as a priming tool, this study also found inconsistencies between the neurophysiological and functional response to priming. Consequently, the mechanisms that underpin the functional effects of priming with iTMS remain unclear, which limit application of this approach.

Within the current study, we sought to address this limitation by using TMS in conjunction with electroencephalography (TMS-EEG). Recent work from our group suggests that the TMS-evoked EEG potential (TEP) can reveal central effects of iTMS which are not indexed by motor evoked potentials (MEPs)(Sasaki et al. 2023). We therefore reasoned that the TEP may be able to provide some additional neurophysiological insight to how iTMS influences motor learning. Consequently, TEPs were recorded before and after practice of a novel visuomotor adaptation task, either in isolation or following application of real or control iTMS.

## Methods

### Participants

Sample size calculations were performed using simulation-based power estimations on Rstudio (Posit-team 2023), using *lme4* (Bates et al. 2015) and *simr* (Green and MacLeod 2016) and were based on our recent study investigating the response to iTMS in M1 (Hand et al. 2023). This study revealed a fixed effects coefficient of -0.306 for the intervention that was used as an unstandardised measure of effect size (Green and MacLeod 2016). This value was lowered by 15% (-0.260) to account for biases in power calculations derived from experimental data (Green and MacLeod 2016) and revealed a required sample size of 16 participants to detect a small-moderate effect of iTMS, given α = 0.05 and 1 – β > 0.85 (~ 0.88). Consequently, 16 healthy young adults (7 men and 9 women; mean age ± SD = 26.1 ± 5.1 years; age range = 19–35 years) were recruited from the University and wider community to participate in the study. All participants were right-handed, free of neurological and psychiatric disorders, were not taking any drugs that influence the central nervous system and had normal or corrected-to-normal vision. Contraindications to TMS were assessed using the TMS adult safety screen (Rossi et al. 2009). A nominal payment of $15 per hour was offered to compensate for time and cost of participation. Written informed consent was provided prior to inclusion and the study was conducted in accordance with the *Declaration of Helsinki.* All experimental procedures were approved by the University of Adelaide Human Research Ethics Committee (approval number: H-026-2008).

### Experimental Arrangement

**Fig. 1:**
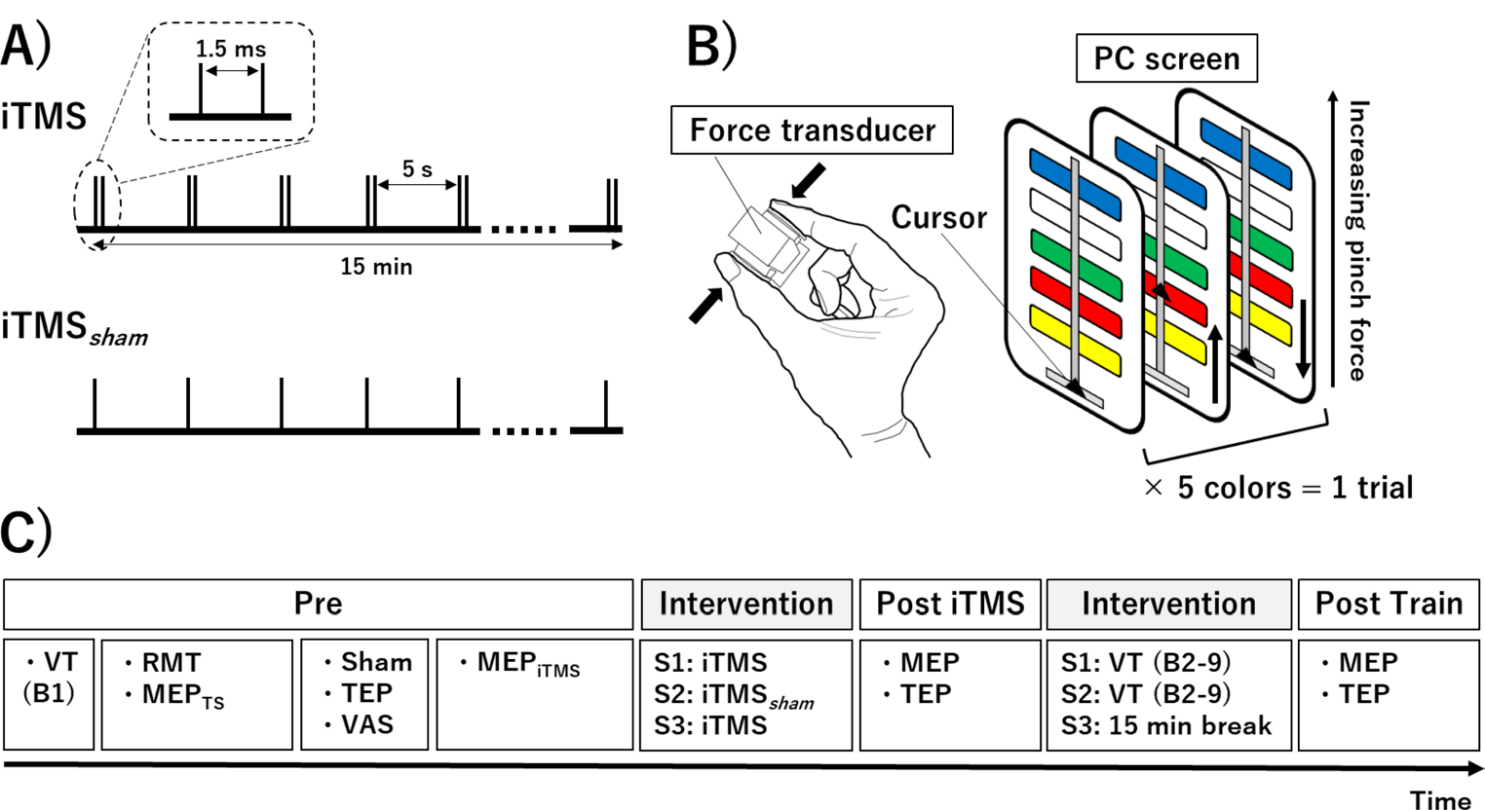
Intervention settings and experimental protocol. (**A**) iTMS intervention parameters. (**B**) Visuomotor training setup and requirements. (**C**) Experimental protocol. Three experimental sessions were performed involving different combinations of iTMS (S1: iTMS; S2: iTMS_Control_; S3: iTMS) and VT (S1: VT; S2: VT; S3: 15 min break). Cortical excitability indexed with both MEPs and TEPs was recorded before iTMS (Pre), immediately after iTMS (Post iTMS) and immediately after VT (Post Train). Abbreviations; B, block; iTMS_Control_, control I-wave periodicity repetitive transcranial magnetic stimulation; iTMS, I-wave periodicity repetitive transcranial magnetic stimulation; MEP, motor-evoked potential; MEP_iTMS_, MEP amplitude producing a response of ~ 0.5–1 mV by iTMS; MEP_TS_, MEP amplitude producing a response of ~ 0.5–1 mV by single pulse TMS; S, session; TEP, transcranial magnetic stimulation-evoked potential; TMS, transcranial magnetic stimulation; VT, visuomotor task

All participants attended three experimental sessions that were each approximately 3.5 h long, held at the same time of day and separated by at least one week (Fig. 1). Each session involved recording MEPs and TEPs before (Pre) and immediately after iTMS (Post iTMS), as well as after visuomotor training (VT)(Post Train). Sessions included real iTMS and VT (iTMS + VT), control iTMS and VT (iTMS_Control_ + VT) and iTMS only (iTMS), with the order of sessions randomized within a participant. For each session, participants sat in a comfortable chair with their right hand pronated on a table and were instructed to keep their eyes open and remain relaxed. Surface electromyography (EMG) was recorded from the right first dorsal interosseous (FDI) muscle via disposable Ag/AgCl electrodes in a belly − tendon montage, with an additional Ag/AgCl electrode placed over the right ulnar styloid as an earth electrode. EMG data were sampled at 2000 Hz using a CED1401 interface (Cambridge Electronic Design, Cambridge, UK), amplified (1000×) and band-pass filtered (20–1000 Hz) by a CED1902 signal conditioner (Cambridge Electronic Design, Cambridge, UK). Line noise was removed using a Humbug mains eliminator (Quest Scientific, North Vancouver, Canada) and recordings were stored on a personal computer for off-line analysis.

### TMS

Monophasic TMS pulses were delivered to the hand area of the left M1 using a figure-of-eight branding iron coil connected to two Magstim 200^2^ stimulators via a Bistim unit (Magstim, Dyfed, UK). The coil was held tangentially to the scalp at an angle of approximately 45° to the sagittal plane, at the location producing the largest stable response in the resting right FDI muscle with a posterior–anterior coil orientation. This position was co-registered to the MNI-ICBM152 brain template (Fonov et al. 2011) using a Brainsight neuronavigation system (Rogue Research Inc, Montreal, Canada). TMS was applied at a rate of 0.25 Hz for MEP and TEP measures with a 10% jitter between trials. Resting motor threshold (RMT) was defined as the minimum intensity needed to evoke MEPs ≥ 50 µV in 5 of 10 consecutive trials during relaxation of the right FDI muscle (Rossini et al. 2015). TMS intensity was expressed as a percentage of maximum stimulator output (%MSO). The test stimulus (TS) for MEP measures was set at the intensity required to produce an MEP of ~ 0.5–1 mV (MEP_TS_) when averaged over 15 trials.

#### iTMS

iTMS involved 180 pairs of stimuli applied every 5 s, resulting in a total intervention time of 15 min (Opie et al. 2018, 2021). The intensity was the same for both stimuli (Sasaki et al. 2022), and was adjusted so that paired stimulation produced a response amplitude of ~ 0.5–1 mV (MEP_iTMS_) assessed over 15 trials before the intervention. An interstimulus interval of 1.5 ms (corresponding to I-wave periodicity) was used. In addition, a control intervention not expected to modulate cortical excitability (single-pulse TMS for 15 min; iTMS_*Control*_) was applied in a separate session. To avoid coil heating during the intervention, ice packs were always used to cool the coil prior to and during iTMS application. This ensured that the same coil could be used for all TMS measures.

### EEG

EEG data was recorded using a WaveGuard EEG cap (ANT Neuro, Hengelo, The Netherlands), with 62 sintered Ag/AgCl electrodes in standard 10–10 positions, connected to an eego mylab amplifier (ANT Neuro, Hengelo, The Netherlands). For all recordings, CPz was used as the reference and AFz was used as the ground. Signals were filtered online (DC–0.26 × sampling frequency), digitized at 8 kHz, and stored on a personal computer for offline analysis. The impedance of all electrodes was constantly kept < 10 kΩ through the experiment. TEPs were recorded in a single block of stimulation that involved 100 pulses set at an intensity of 100% RMT. During all EEG recordings, participants listened to white noise played through in-canal earphones, with ear defenders (Peltor Optime, 3 M; 34db reduction) to minimize the influence of auditory-evoked potentials. The volume of auditory masking was individually adjusted to minimize audition of the TMS click (Biabani et al. 2019; Rocchi et al. 2021).

### Visuomotor Training

A sequential visual isometric pinch task (SVIPT) was used to assess motor skill acquisition (Opie et al. 2020; Hand et al. 2021, 2023). Before the task, maximum voluntary contraction (MVC) force was assessed by a force transducer. Participants grasped the transducer between the right index finger and thumb for 3–5 s (repeated three times). The highest force value was set as MVC. During the task, the position of a digital cursor was manipulated by a participant using the pinch grip, with the aim of a single trial being to accurately move the cursor between 5 color targets in a specific order (consistent within each session) while returning to baseline (0% MVC) between each color. The coloured targets disappeared at the end of each trial and reappeared for the start of the next trial. To increase task difficulty and reduce carry-over of learning between sessions, a non-linear transform was used to relate force application to cursor movement. Specifically, logarithmic, exponential and sigmoidal transforms were used for the iTMS + VT, iTMS_*Control*_ + VT and iTMS sessions, respectively. A baseline block involving 6 trials (Pre) was completed prior to TMS measures. VT then involved 8 blocks of 8 trials (B1–B8). Participants completed each trial at their own pace, but they were instructed to focus on improving their speed and accuracy during each trial. A ‘skill’ score (see below) was calculated at the end of each block and displayed on a screen to provide feedback on performance.

### Data Analysis

#### MEP Data

MEP data were inspected visually and trials with muscle activity > 20 µV peak-to-peak amplitude in the 100 ms prior to TMS were rejected. MEP amplitude recorded in each trial was then quantified peak-to-peak and expressed in millivolts (mV). MEP amplitudes recorded during iTMS were averaged over 10 consecutive stimuli, resulting in a total of 18 blocks.

#### VT Data

Skill scores were calculated for each block based on the movement speed and accuracy. Speed was measured by the average movement time (MT) for each trial. Accuracy was defined based on the error between the applied force and the force required to meet the center of the target. This was calculated for each of the 5 force peaks within a trial using the Euclidean distance, and then averaged over peaks to produce a trial error score. Skill scores were finally calculated using the following formula, as proposed by Reis et al. (2009).

$$\:Skill=\frac{(1-error)}{error\:\left({ln\:\left(movement\:time\right)}^{b}\right)}$$

The dimensionless free b parameter has been shown to be insensitive to changes in performance, and thus was set at a consistent 1.627 (Stavrinos and Coxon 2017).

#### EEG Data

All preprocessing and subsequent analysis was performed according to previously reported procedures (Rogasch et al. 2017; Mutanen et al. 2020; Sasaki et al. 2022) using custom scripts on the MATLAB platform (R2019b, Mathworks, USA), in addition to EEGLAB (v2020.0) (Delorme and Makeig 2004), TESA (v1.1.1.) (for review, see Rogasch et al. 2017) and Fieldtrip (v20200607) (Oostenveld et al. 2011) toolboxes. Data were epoched from − 1500 ms to 2000 ms around the TMS trigger, baseline corrected from − 500 ms to -5 ms and merged into a single file including all time points (Pre, Post iTMS, and Post Train). Channels demonstrating persistent, large amplitude muscle activity or noise were manually removed, and the peak of the TMS artifact was removed by cutting the data from − 2 to 10 ms and replacing it using cubic interpolation. The data was subsequently downsampled from 8 kHz to 500 Hz and epochs demonstrating bursts of muscle activity or electrode noise were manually removed. Interpolated data from − 2 to 10 ms was then replaced with constant amplitude data (i.e., 0 s), avoiding the addition of information prior to ICA (Rogasch et al. 2017). An initial independent component analysis (ICA) was then run using the FastICA algorithm (Hyvarinen and Oja 2000), and a couple of independent components (IC’s) representing the tail of the TMS-evoked muscle artifact were removed (for review, see Rogasch et al. 2017). Constant amplitude data from − 2 to 10 ms were then replaced with cubic interpolation prior to the application of band-pass (1–100 Hz) and notch (48–52 Hz) filtering (zero-phase 4th order Butterworth filter implemented). In order to remove any additional decay artifacts still present after the first round of ICA, the source-estimate-utilizing noise-discarding (SOUND) algorithm was then applied; this approach estimates and removes artefactual components within source space, and also allows missing electrodes to be estimated and replaced (Mutanen et al. 2018). A regularization parameter of 0.1 was used and 5 iterations were completed. Following SOUND, data around the TMS pulse were again replaced with constant amplitude data prior to application of a second round of ICA, and IC’s associated with blinks, eye movements, electrode noise, and muscle activity were automatically identified using the TESA compselect function (default settings) and visually inspected prior to removal (for review, see Rogasch et al. 2017). Data around the TMS pulse were then replaced with cubic interpolation, and all channels were re-referenced to average prior to a final baseline correction (-500 ms to -5 ms).

### Statistical Analysis

An initial analysis was applied in the frequentist framework, and identified several negative findings that were inconsistent with expected effects of iTMS. To better understand these outcomes, we therefore completed a supplementary analysis wherein all data were re-analysed within the Bayesian framework. This allowed us to quantify the extent of evidence in favour of the null hypothesis, and to indicate situations in which there was insufficient evidence to accept or reject the null hypothesis. While the initial frequentist analysis is described below, details of the Bayesian analysis can be found in supplementary materials.

All frequentist analyses were performed using PASW statistics software version 28 (SPSS; IBM, Armonk, NY, USA) or Fieldtrip toolbox (EEG data only). All data were assessed using generalized linear mixed models (GLMM). Data distribution was initially assessed using Kolmogorov-Smirnov tests and Q-Q plots (Lo and Andrews 2015; Puri and Hinder 2022). These identified that VAS (all items) and iTMS intensity were normally distributed and could therefore be fit with a Gaussian distribution (i.e., linear mixed model). However, other TMS intensities, MEP amplitude, and VT data all showed negatively skewed distributions and were therefore modelled using a Gamma distribution with identity link function (Lo and Andrews 2015). Each model involving MEP responses (raw MEP amplitude) used individual trial data, whereas all models included the maximal participant random effects structure. Model fit was assessed using the Akaike’s Information Criterion (AIC). Post hoc analysis of all significant main effects and interactions were performed using custom contrasts with Bonferroni correction, and significance was set at *P* < 0.05. All data are presented as estimated marginal means (EMM) and 95% confidence intervals (95% CI).

#### MEP Data

One-factor GLMM analysis with repeated measures (GLMM_RM_) was used to compare baseline RMT, TS intensity, iTMS intensity, MEP_TS_, and MEP_*iTMS*_ between sessions (iTMS + VT, iTMS_*Control*_ + VT, and iTMS). For TS MEP amplitudes before and after interventions, two-factor GLMM_RM_ was used to compare values between sessions and time points (Pre, Post iTMS, and Post Train). Two-factor GLMM_RM_ was also used to compare MEP amplitudes during iTMS between sessions and blocks (B1–B18).

#### VT Data

One-factor GLMM_RM_ was used to compare baseline error, MT, and skill between sessions (iTMS + VT, iTMS_*Control*_ + VT, and iTMS). Two-factor GLMM_RM_ was also used to compare error, MT, and skill between sessions (iTMS + VT and iTMS_*Control*_ + VT) and blocks (Pre, B1–B8).

#### TEP Data

For data within each session, TEPs were compared between Pre and Post iTMS, Pre and Post Train, or Post iTMS and Post Train using cluster-based non-parametric permutation analysis utilising *t*-statistics. Cluster-based *t*-statistics were also used to compare baseline TEPs between sessions (i.e., iTMS + VT vs. iTMS, iTMS_*Control*_ + VT vs. iTMS, iTMS + VT vs. iTMS_*Control*_ + VT. Clusters were defined as two or more neighboring electrodes (defined using the electrode layout for the 64 channel WaveGuard caps) and 10,000 iterations were applied. A cluster was deemed significant if the cluster statistic exceeded *P* < 0.05 when compared with the permutation distribution. A growing body of literature suggests that early TEP components (i.e., < 50 ms post-TMS) are likely to be less confounded by indirect brain responses generated by sensory input, and more likely to reflect responses generated by direct activation of the brain by TMS (Biabani et al. 2019, 2023; Conde et al. 2019; Gordon et al. 2021; Rocchi et al. 2021). Consequently, comparisons between conditions with the current study were limited to the early TEP components, including N15 (10–15 ms), P30 (25–35 ms) and N45 (40–50 ms).

## Results

All 16 participants completed the 3 sessions without any adverse events (mean time between sessions ± SD: S1–S2, 9.6 ± 3.7 days; S2–S3, 12.0 ± 8.0 days). A total of 2.8% and 5.8% of trials were removed from TS MEP and during iTMS MEP, respectively. While the results of the frequentist analysis are detailed below, results from Bayesian analyses can be found in supplementary materials. Baseline characteristics for MEP and VT are compared between sessions in Table 1. No difference was found between sessions for RMT or TS intensity (Session effect: RMT, *F*_(2,45)_ = 2.747, *P* = 0.075; TS, *F*_(2,45)_ = 0.189, *P* = 0.828), whereas iTMS intensity did vary between sessions (Session effect: *F*_(2,45)_ = 37.331, *P* < 0.001 with higher intensity for iTMS_*Control*_ + VT than other sessions (*F*_(2,45)_ = 37.366, *P* < 0.001). Baseline TS and iTMS MEP amplitudes showed no differences between sessions (Session effects: MEP_TS_, *F*_(2,690)_ = 0.362, *P* = 0.697; MEP_iTMS_, *F*_(2,477)_ = 1.593, *P* = 0.204). Furthermore, comparisons of baseline error, MT, and skill showed no differences between sessions (Session effects: Error, *F*_(2,284)_ = 1.763, *P* = 0.173; MT, *F*_(2,281)_ = 0.010, *P* = 0.990; Skill, *F*_(2,281)_ = 1.751, *P* = 0.176).

**Table 1:** Baseline characteristics, corticospinal responses, and motor skills for each session

	iTMS + VT	iTMS_Control_ + VT	iTMS
RMT (%MSO)	60.1 [55.9, 64.1]	60.9 [56.9, 65.0]	60.4 [56.1, 64.2]
TS (%MSO)	72.6 [67.2, 78.0]	72.5 [67.1, 77.9]	72.3 [66.8, 77.6]
iTMS (%MSO)	63.6 [59.7, 67.3]	64.8 [61.2, 68.7]	63.9 [60.0, 67.5]
MEP_TS_ (mV)	0.73 [0.61, 0.87]	0.70 [0.59, 0.82]	0.68 [0.58, 0.81]
MEP_iTMS_ (mV)	0.43 [0.29, 0.62]	0.53 [0.41, 0.70]	0.61 [0.42, 0.91]
Error (a.u.)	0.18 [0.15, 0.23]	0.22 [0.18, 0.28]	0.25 [0.20, 0.31]*
MT (sec)	3.32 [2.89, 3.89]	3.34 [2.90, 3.84]	3.31 [2.86, 3.86]
Skill (a.u.)	4.21 [2.94, 5.97]	3.40 [2.55, 4.58]	3.28 [2.44, 4.57]

EMM [95% CI; lower, upper]. *P < 0.05 compared to iTMS_*Control*_ + VT. Abbreviations: iTMS_*Control*_, control I-wave periodicity repetitive transcranial magnetic stimulation; iTMS, I-wave periodicity repetitive transcranial magnetic stimulation; MEP, motor-evoked potential; MEP_iTMS_, MEP amplitude producing a response of ~ 0.5–1 mV by iTMS; MEP_TS_, MEP amplitude producing a response of ~ 0.5–1 mV by single pulse TMS; %MSO, %maximum stimulator output; MT, movement time; RMT, resting motor threshold; TS, test stimulus; VT, visuomotor task

### Effects of iTMS on Corticospinal Excitability

**Fig. 2:**
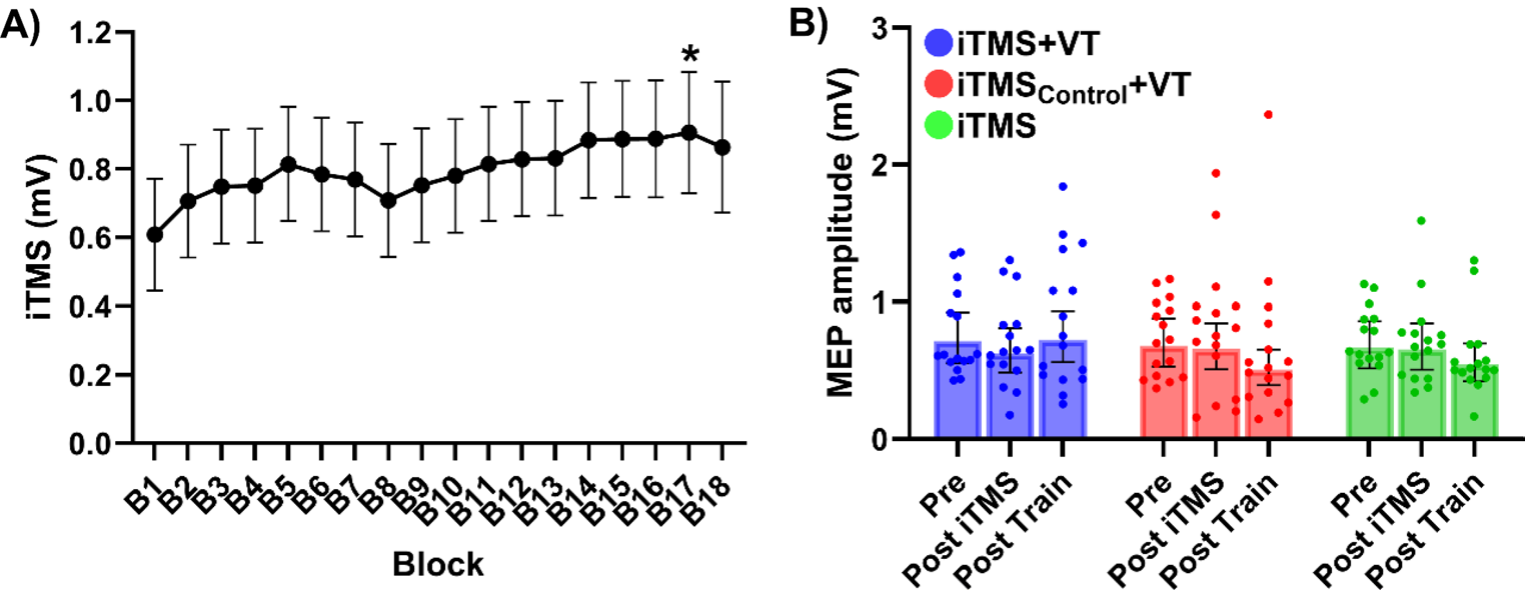
Corticospinal excitability changes by iTMS and VT. (**A**) MEP amplitudes during iTMS, averaged over 10 consecutive MEP trials. (**B**) TS MEP amplitudes before and after iTMS and VT. *P < 0.05 compared to B1. EMM ± 95% CI. *Abbreviations*; B, block; iTMS_Control_, control I-wave periodicity repetitive transcranial magnetic stimulation; iTMS, I-wave periodicity repetitive transcranial magnetic stimulation; MEP, motor-evoked potential; VT, visuomotor task

Figure 2A shows changes in MEP amplitude during iTMS. No difference was found between sessions (Session effect: *F*_(2,8085)_ = 1.054, *P* = 0.349), and there was no interaction between factors (Session x Block interaction: *F*_(34,8085)_ = 0.739, *P* = 0.865). However, values varied over blocks (Block effect: *F*_(17,8085)_ = 1.881, *P* = 0.015), with post-hoc comparisons showing increased amplitude during block 17 relative to block 1 (*P* = 0.049). TS MEP amplitudes before and after iTMS and VT are shown in Fig. 2B. MEP amplitudes were not different between sessions (Session effect: *F*_(2,2090)_ = 0.554, *P* = 0.575) or time points (Time effect: *F*_(2,2090)_ = 1.557, *P* = 0.211) and there was no interaction between factors (Session x Time interaction: *F*_(4,2090)_ = 1.251, *P* = 0.287).

### Effects of iTMS on Visuomotor Training

**Fig. 3:**
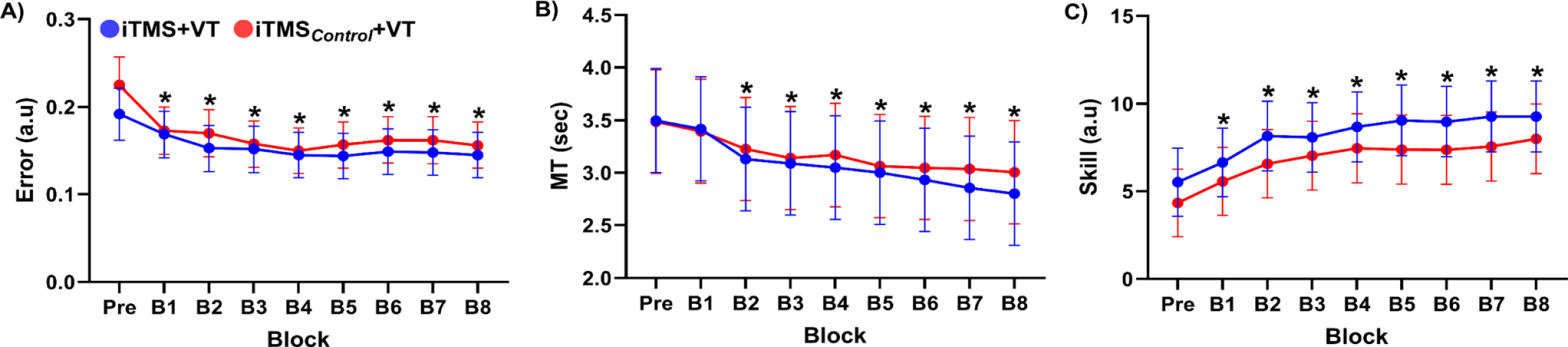
Changes in motor skills over blocks. Panels (**A**, **B**, **C**) represent error, MT, and skill before and after iTMS, respectively. *P < 0.05 compared to Pre. EMM ± 95% CI. Abbreviations; B, block; iTMS_Control_, control I-wave periodicity repetitive paired-pulse transcranial magnetic stimulation; iTMS, repetitive I-wave periodicity paired-pulse transcranial magnetic stimulation; MT, movement time; VT, visuomotor task

Performance during VT is shown in Fig. 3. Error was not different between sessions (Session effects: *F*_(1,2219)_ = 1.923, *P* = 0.166), and there was no interaction between factors (Session x Block interaction: *F*_(8,2219)_ = 0.343, *P* = 0.949). However, error varied over blocks (Block effect: *F*_(8,2219)_ = 3.613, *P* < 0.001), with *post-hoc* comparisons showing decreased error during training (i.e., block 1–8) relative to baseline (all *P* < 0.02)(Fig. 3A). MT was not different between sessions (Session effect: *F*_(1,2198)_ = 0.828, *P* = 0.363) and there was no interaction between factors (Session x Block interaction: *F*_(8,2198)_ = 0.768, *P* = 0.631). However, MT varied over blocks (Block effect: *F*_(8,2298)_ = 19.806, *P* < 0.001), with *post-hoc* comparisons showing decreased MT during block 2–8 relative to Pre (all *P* < 0.001)(Fig. 3B). Furthermore, skill varied between sessions (Session effect: *F*_(1,2205)_ = 6.044, *P* = 0.014), with *post-hoc* comparisons showing greater skill for iTMS + VT relative to iTMS_*Control*_ + VT (*P* = 0.014). Skill also varied over blocks (Block effect: *F*_(8,2205)_ = 26.844, *P* < 0.001), with *post-hoc* comparisons showing increased skill during block 1–8 relative to Pre (all *P* < 0.002). However, there was no interaction between factors (Session x Block interaction: *F*_(8,2205)_ = 0.390, *P* = 0.926).

Given the differences in skill across blocks that included the baseline timepoint, the analysis of motor performance measures was repeated using data that were expressed as a percentage of baseline. Using this approach, Error was no longer different between blocks (Block effect:

*F*_(7,2029)_ = 0.784, *P* = 0.600), whereas skill was no longer different between sessions (Session effect: *F*_(1,2016)_ = 2.587, *P* = 0.108). All other results were consistent with the original analysis of non-normalised data.

### TEPs

**Fig. 4:**
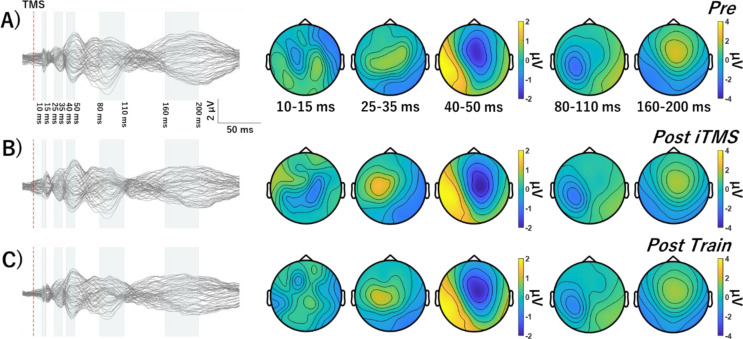
Grand average TEP waveforms and topographies in iTMS + VT session. TEP Response to M1 stimulation before (**A**) and after iTMS (**B**) and VT (**C**). Waveforms show several typical TEP components, named N15, P30, P45, N100, and P180. Abbreviations; TMS, transcranial magnetic stimulation; iTMS, I-wave periodicity repetitive paired-pulse transcranial magnetic stimulation

**Fig. 5:**
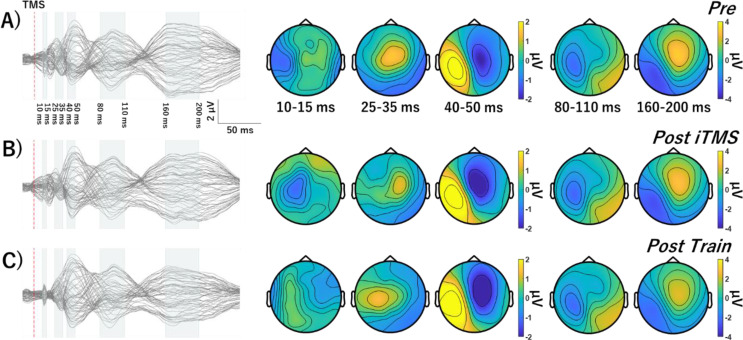
Grand average TEP waveforms and topographies in iTMS_*Control*_ + VT session. Response to M1 stimulation before (**A**) and after iTMS_*Control*_ (**B**) and VT (**C**). Waveforms show several typical TEP components, named N15, P30, P45, N100, and P180. Abbreviations: iTMS_*Control*_, control I-wave periodicity repetitive paired-pulse transcranial magnetic stimulation; TMS, transcranial magnetic stimulation

**Fig. 6:**
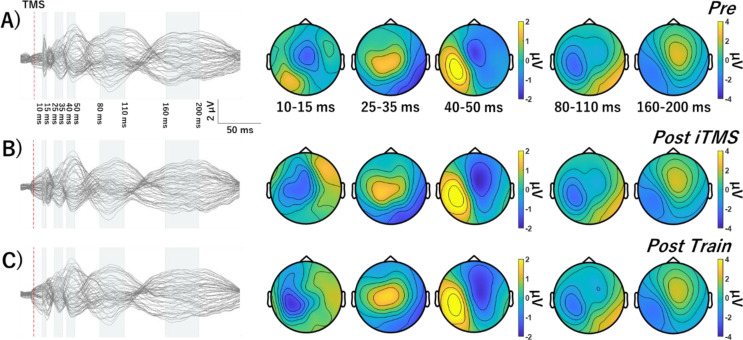
Grand average TEP waveforms and topographies in iTMS session. Response to M1 stimulation before (**A**) and after iTMS (**B**) and 15 min break (**C**). Waveforms show several typical TEP components, named N15, P30, P45, N100, and P180. Abbreviations: TMS, transcranial magnetic stimulation; iTMS, I-wave periodicity repetitive paired-pulse transcranial magnetic stimulation

**Fig. 7:**
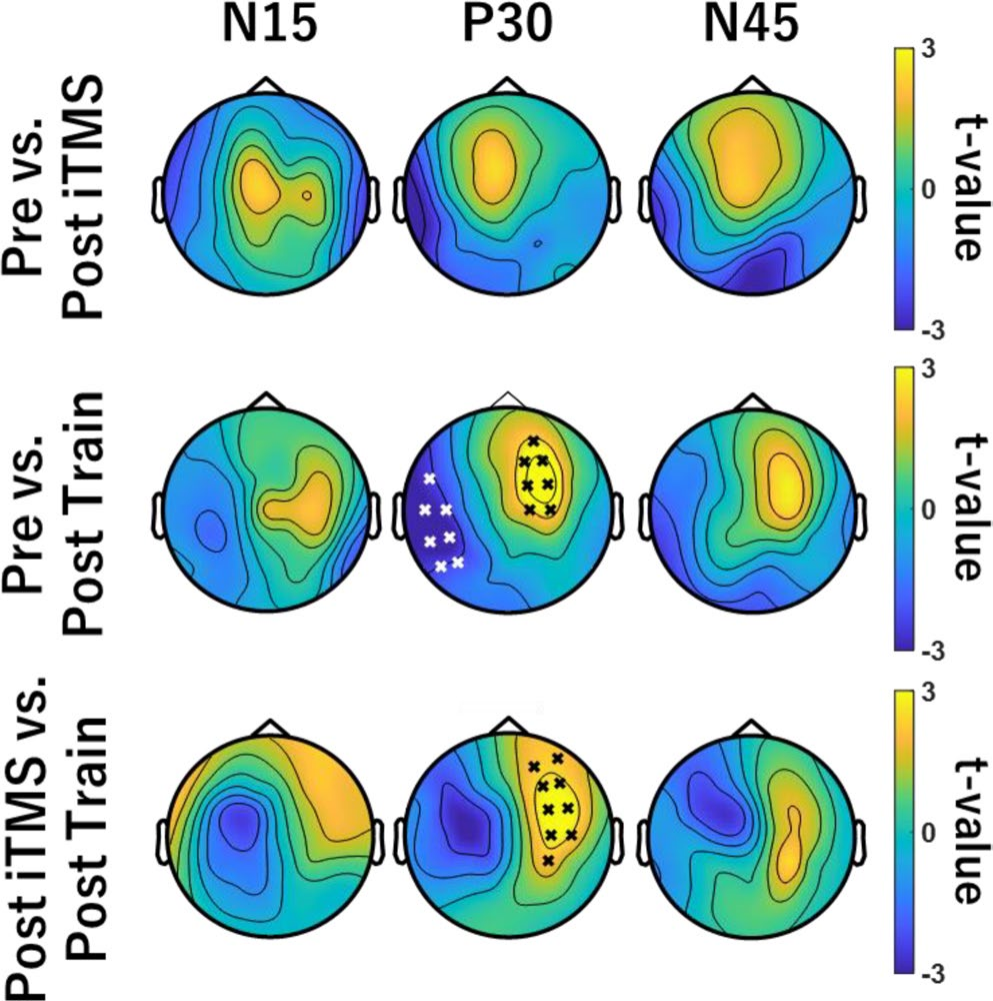
Comparison of TEPs between Pre and Post in iTMS_*Control*_ + VT session. These topographies represent cluster-based permutation *t*-test comparing the TEPs amplitudes before and after iTMS_*Control*_ (top row), before iTMS_*Control*_ and after VT (middle row), after iTMS_*Control*_ and after VT (bottom row). Black and white crosses show significant clusters between Pre- and Post Train- or Post iTMS- and Post Train-P30 amplitude

The average number of channels, epochs and IC’s removed during each step of the preprocessing pipeline are shown in Table 2. Figures 4, 5 and 6 show grand-average TEP waveforms elicited by M1 stimulation. Baseline TEP components were not different between sessions (all *P* > 0.08). For each iTMS + VT and iTMS session, there were no differences between time points (all *P* > 0.15). For iTMS_*Control*_ + VT, comparisons of P30 between Pre and Post Train identified significant negative (*P* = 0.028) and positive clusters *(P* = 0.042). Comparisons of P30 between Post iTMS and Post Train also identified a significant positive cluster (*P* = 0.033) (Fig. 7). However, no differences were found for the N15 and N45 (all *P* = 1).

**Table 2:** Number of channels, epochs, and independent components removed during cleaning of TEPs

	iTMS + VT	iTMS_Control_ + VT	iTMS
Channels (pre)	0.38 ± 0.17	0.38 ± 0.21	0.44 ± 0.23
Channels (Post iTMS)	0.38 ± 0.17	0.38 ± 0.21	0.44 ± 0.23
Channels (Post Train)	0.38 ± 0.17	0.38 ± 0.21	0.44 ± 0.23
Epoch (pre)	1.94 ± 0.42	2.13 ± 0.48	1.75 ± 0.55
Epoch (Post iTMS)	4.06 ± 1.14	2.25 ± 0.48	2.00 ± 0.49
Epoch (Post Train)	1.88 ± 0.75	2.81 ± 1.09	2.13 ± 0.87
ICA1 (TS)	2.75 ± 0.40	2.56 ± 0.44	2.75 ± 0.43
ICA2 (TS)	7.25 ± 0.79	7.63 ± 0.81	7.13 ± 0.82

EMM [95% CI; lower, upper]. Abbreviations; iTMS_*Control*_, control I-wave periodicity repetitive paired-pulse transcranial magnetic stimulation; iTMS, I-wave periodicity repetitive paired-pulse transcranial magnetic stimulation; ICA, independent component analysis; TS, test stimulus; VT, visuomotor task

## Discussion

Within the current study, we aimed to further characterise the neurophysiological processes that underpin beneficial effects of iTMS on motor learning. To achieve this, TEPs were recorded before and after a visuomotor adaptation task that was practiced in isolation or following application of iTMS. While skill increased in response to training, the magnitude of this effect was not different between priming conditions, suggesting that iTMS was ineffective as a priming intervention. However, iTMS also failed to induce the expected potentiation of MEP amplitude, complicating interpretation of the response to training. Despite this, differential effects on TEP amplitude suggested that training produced changes in cortical activity that were cancelled by priming.

### Skill Acquisition, Corticospinal Excitability and Priming

Previous work has reported that, when applied prior to training, a neuromodulatory NIBS intervention can improve acquisition of a novel motor skill (e.g., Jung and Ziemann 2009). Within this construct, NIBS-dependent modulation of motor network activity is thought to generate a neural environment that is more amenable to the neuroplastic changes required to learn new patterns of motor behaviour (Müller-Dahlhaus and Ziemann 2015). This has been supported by studies showing that priming-dependent modulation of motor learning is accompanied by related changes in motor cortical excitability (Ziemann et al. 2004; Jung and Ziemann 2009). Within the current study, visuomotor training resulted in improved skill levels that are consistent with previous work from our group (Opie et al. 2020; Hand et al. 2021, 2023) and others (Reis et al. 2009; Ho et al. 2022). While skill was significantly greater in the iTMS + VT condition, examination of normalised data showed this stemmed from baseline differences in performance (see *Results* and Fig. 3C). In addition, MEP measures of corticospinal excitability were also unchanged by priming or training. While these results could suggest an inability of iTMS to influence skill acquisition or corticospinal excitability (when assessed with MEPs), this outcome is inconsistent with several previous studies from our group (Opie et al. 2018, 2021; Sasaki et al. 2023) and others (Thickbroom et al. 2006; Cash et al. 2009; Sewerin et al. 2011). An alternative explanation could be that, despite sample size calculations based on our previous work (see above), and a sample size that is consistent with several previous studies that reported facilitatory effects of iTMS (Shirota et al. 2016; Long et al. 2017; Opie et al. 2021), the current study may have been underpowered. To examine this possibility, we completed a supplementary analysis of our data, with all comparisons being repeated within the Bayesian framework. The results of this analysis suggested that effects of iTMS on both MEPs and training were inconsistent and failed to provide sufficient evidence to reject or accept the null hypothesis. Consequently, it seems that iTMS was less effective within the current study, and our sample was therefore underpowered for detecting associated changes in MEPs and skill.

As suggested above, we recently reported significant effects of iTMS on both MEPs and skill acquisition during SVIPT, providing the impetus for the current studies further examination with TEPs. Given that the current study applied methodology comparable to this previous work (including the same research environment and protocols), the extent of the divergence in results is surprising. A minor discrepancy between the studies was that the iTMS ISI differed by 0.1 ms, possibly contributing to variability. However, it can be expected that the timing of I-waves within individual participants varied by more than 0.1 ms (Sewerin et al. 2011). Consequently, it seems that the minor difference in ISI between studies would explain less variance than can be accounted for by the fixed ISI (relative to I-wave timing within individuals), and certainly wouldn’t account for the divergent findings of these studies. A more likely explanation is that the results reported here further demonstrate the variability that is being increasingly recognised within the field, particularly with respect to replication of canonical effects. For example, there is a growing literature that reports negative findings with respect to the effects of both neuromodulatory interventions (Hamada et al. 2013; López-Alonso et al. 2014; Wiethoff et al. 2014; Jonker et al. 2021) and motor training (Bestmann and Krakauer 2015) on MEP amplitude, in addition to the effect of priming stimulation on motor learning (Lopez-Alonso et al. 2018; Sasaki et al. 2018).

The factors driving this variability are likely to be multifactorial; these have been covered in detail elsewhere (Ridding and Ziemann 2010), but are known to include attention, cortisol levels (Sale et al. 2007, 2008), genetics (Cheeran et al. 2008), physical activity (Cirillo et al. 2009), chronotype (Salehinejad et al. 2021) and neural activity (Zrenner et al. 2022), in addition to the many potential methodological sources of variability (including statistical)(for review, see Guerra et al. 2020). An additional point that the current study can somewhat speak to is the way in which outcomes are assessed. For example, while MEPs were insensitive to the intervention applied here, TEPs were instead altered by training (see below). We do not mean to suggest that TEPs should be considered a superior approach; indeed, these responses are still heavily encumbered by methodological limitations, and their interpretation is being actively developed. Nonetheless, the contrast between findings reported here demonstrates the potential for alternative outcome measures to influence our results.

Control iTMS within the current study involved single-pulse stimulation applied with the same frequency and duration as real iTMS. This approach has been used by previous iTMS studies, which reported no change in MEPs during or after application (Silbert et al. 2011; Teo et al. 2012). In contrast to this, we found an apparent increase in MEP amplitude during application of control iTMS (data not shown). Although inconsistent with previous iTMS studies, other work has shown that there can be cumulative effects of single-pulse TMS over a period comparable to the application of iTMS (Pellicciari et al. 2016). While the specific reason this was apparent in the current but not previous studies remains unclear, it nonetheless demonstrates the need for an improved control paradigm for iTMS. We have previously used control stimulation that involved paired-pulse stimuli with ISIs associated with non-facilitatory periods of the I-wave recruitment profile, the order of which are pseudorandomised between trials (Liao et al. 2022). While this appears to be a promising approach, it has only been applied during application of cerebellar tDCS and will therefore need to be verified during isolated application to M1.

### Effects of Motor Training on Cortical Reactivity are Removed Following iTMS

While N15 and N45 were unchanged in any condition, P30 was found to vary in response to motor training alone (i.e., iTMS_*Control*_ + VT session). Specifically, amplitude was increased and more lateralized over ipsilateral central electrodes (Figs. 5 and 7). While previous work has used TMS-EEG to investigate changes in cortical reactivity associated with visuomotor adaptation (Koch et al. 2020; Taga et al. 2021), effects of learning were limited to the later peaks that are associated with increased contamination from sensory input (Biabani et al. 2019). Consequently, as far as we are aware, the current study is the first to report a modulation of the early TEP peaks following visuomotor training. The P30 has been associated with local excitatory and inhibitory processes (Cash et al. 2017; Sasaki et al. 2021), and its modulation during training is therefore consistent with neural changes driven by motor learning (for review, see Dayan and Cohen 2011). Interestingly, these changes were apparent despite MEPs being unaffected by learning, suggesting that TEP-based measures of cortical reactivity may be a more sensitive index of the neurophysiological response to training. However, it will be important for future work to investigate the test-retest reliability of this outcome to demonstrate its relevance to motor learning.

Whereas training alone resulted in a modulation of the TEP, this effect was removed when training was primed by iTMS. One explanation for this could be that priming stimulation interfered with the neuronal processes recruited by training. We recently reported effects of iTMS on TEPs that would generally be considered as beneficial to the neurophysiological processes associated with learning (i.e., disinhibition of local intracortical circuits; Ziemann et al. 2001; Sasaki et al. 2023) and it is therefore unclear why this would be the case. However, the timing of this disinhibition is likely to be important (Ziemann and Siebner 2008), and its application prior to learning may have resulted in metaplastic effects that interfered with the brains response to training. Nonetheless, these neurophysiological effects failed to influence the functional response to training. A question that stems from this is whether the cortical effects of priming: *(1)* failed to exceed some threshold required to influence learning or *(2)* were not directly relevant to learning/ were not conducive to improving learning. While the former option would suggest that increasing the strength of the priming stimulus (e.g., higher intensities, longer duration, paired priming blocks) may facilitate an impact on learning, the latter may instead imply that different priming would be needed, perhaps targeting other nodes of the motor network. The current study is unable to differentiate between these options and it will be important for future research to investigate them further.

In conclusion, the current study aimed to further investigate the neurophysiological effects of iTMS on cortical excitability and motor learning. Against expectations, the normally robust effects of iTMS on MEP amplitude were absent, training failed to modulate corticospinal excitability, and priming did not influence motor learning. In contrast, the P30 was modulated by motor learning, and this effect was removed when training was preceded by priming iTMS. While this suggests that priming was able to influence the cortical response to training, it remains unclear why this failed to impact learning.

## Electronic Supplementary Material

Below is the link to the electronic supplementary material.

Supplementary Material 1

## Data Availability

Deidentified data can be made available upon reasonable request to the corresponding author.

## Abbreviations

EEG: electroencephalography
EMG: Surface electromyography
EMM: estimated marginal means
FDI: First dorsal interosseous
GLMM: generalised linear mixed models
ICA: independent component analysis
iTMS: Repetitive paired-pulse transcranial magnetic stimulation
M1: primary motor cortex
MEP: motor-evoked potentials
MSO: Maximum stimulator output
MT: movement time
MVC: Maximal voluntary contraction
PAS: Paired-associative stimulation
RMT: Resting motor threshold
TEP: TMS-evoked potential
TMS: Transcranial magnetic stimulation
TS: test stimulus
VT: Visuomotor task
