# Supplementary material for: Investigating the Effects of Repetitive Paired-Pulse Transcranial Magnetic Stimulation on Visuomotor Training Using TMS-EEG
Source: Brain Topogr. 2024 Jul 27;37(6):1158–70. doi: 10.1007/s10548-024-01071-1 (PMC11408544; doi:10.1007/s10548-024-01071-1)
Supplement: Supplementary file 1 — Supplementary Material 1 [file 10548_2024_1071_MOESM1_ESM.docx]

**Supplementary analysis of data using Bayesian methods**

All TMS and behavioral data were analyzed in R version 4.xxx (Team 2023b) using R studio version 2023.12.1+402 (Team 2023a). Bayesian generalized linear mixed models (GLMM) were employed to analyze all TMS and behavioral data using ‘brms’ package (Bürkner 2017). Visual inspection of MEP amplitude and VT data revealed positively skewed distributions. Models investigating MEP amplitude and VT performance measures were therefore fit using Gamma distributions with log link function (Lo and Andrews 2015, Puri and Hinder 2022, Liao *et al.* 2023), and included a maximal effects structure (all intercepts and slopes) (Barr 2013). Models investigating baseline TMS intensities were fitted using normal distribution with identity link, and included only random participant intercepts.

*MEP data*: Three single-factor models were used to investigate the effects of session (iTMS+VT, iTMS*_Control_*+VT, and iTMS) on baseline stimulation intensities for assessment of RMT, TS, and iTMS. A two-factor model was used to investigate the effects of session and block (B1-B18) on MEP amplitude during iTMS, whereas another two-factor model was used to investigate the effects of session and time point (Pre, Post iTMS, and Post Train).

*VT data*: Three single-factor models were used to investigate the effects of session on baseline error, MT and skill. Three two-factor models were used to investigate the effects of session (iTMS+VT and iTMS*_Control_*+VT) and block (Pre, B1–B8) on error, MT and skill during each session.

All posterior distributions were estimated using the No-U-Turn-Sample (NUTS) extension of Hamiltonian Monte Carlo Markov Chain (MCMC) algorithm. Each model was run using 4 independent chains with 1000 warm up and 3000 post-warm up samples (totaling 12,000 post-warm up samples). Furthermore, while iTMS has been shown to effectively potentiate M1 excitability (Thickbroom *et al.* 2006, Cash *et al.* 2009) and modulate visuomotor performance (Hand *et al.* 2023), meta-analysis of this technique has reported a high risk of bias within early studies (*n* = 11) (Kidgell *et al.* 2016). We therefore represented this uncertainty in the posterior distributions using a weakly informative prior with normal distribution (*N* ~ 0,1). Model chain convergence was assessed by ensuring Rhat vales were < 1.1 and visual inspection of post-warm up samples (Gelman and Rubin 1992). Posterior predictive checks were conducted to ensure simulated data closed matched observed data (Gabry *et al.* 2019).

Following model fitting, the ‘emmeans’ package (Lenth 2023) was used to generate custom contrasts probing main effects and interactions. We indexed effect *existence* (i.e., consistency of the effect) using the probability of direction (*pd*), which reports the proportion of the posterior with the same sign as the median and ranges from 50% to 100 % (Makowski *et al.* 2019). This measure is correlated with the frequentist *P*-value (Makowski et al. 2019), and *pd* > 95% indicates a consistent effect. We assessed effect *significance* (i.e., magnitude of the effect) using the region of practical equivalence (ROPE), which measures how much posterior distributions overlapped a pre-defined range centered on zero (Makowski et al. 2019). Within the present study, the ROPE range was defined as ±5% SD of each model (Kruschke 2018) and was used to decide the null hypothesis of no difference. For each contrast, the null hypothesis was accepted if the 89% high density interval (HDI) of each posterior distribution fell completely within ROPE (100% in ROPE) or rejected if it fell completely outside of the ROPE (0% in ROPE), whereas no decision was made if the 89% HDI partially overlapped the ROPE (Opie *et al.* 2023, Puri *et al.* 2023). Unless stated otherwise, all MEP and VT data are presented as median and 89% HDI.

**Results**

MEP amplitude during the first block of iTMS alone was consistently increased relative to iTMS+VT (*pd* = 95.0%), but this failed to reach a practical level of significance (2.2% in ROPE). Baseline error was increased during iTMS compared to iTMS+VT session, with this effect being consistent (*pd* = 97.7%) and significant (0% in ROPE). MT did not vary at baseline between iTMS+VT and iTMS*_Control_* +VT (*pd* = 64.4%, 100% in ROPE). All other comparisons for baseline stimulation intensities, MEP amplitude, and VT were inconsistent (*pd* = 53.8%-94.7%) and there was insufficient evidence to accept or reject the null hypothesis (2.2%-99.3% in ROPE).

*Effects of iTMS on corticospinal excitability.*

MEP amplitude during and following iTMS are presented in figure 2. MEP amplitude during iTMS gradually increased throughout the intervention (*pd* = 95-100% and 0% in ROPE for consistent and significant effects). Furthermore, while there was insufficient evidence to accept or reject the null hypothesis for the effects of session on MEP amplitude during blocks 1-17 (*pd* = 48.0%-86.9%, 13.9%-20.7% in ROPE), responses during block 18 of iTMS*_Control_*+VT were increased relative to iTMS+VT, with this effect being consistent (*pd* = 97.1%) and significant (0% in ROPE; Fig. 2A). In contrast, there was insufficient evidence to accept or reject the null hypothesis for effects of session and time on single-pulse MEP amplitude following iTMS (*pd* = 50.2%-96.4%, 0.9%-50.1% in ROPE; Fig. 2B).


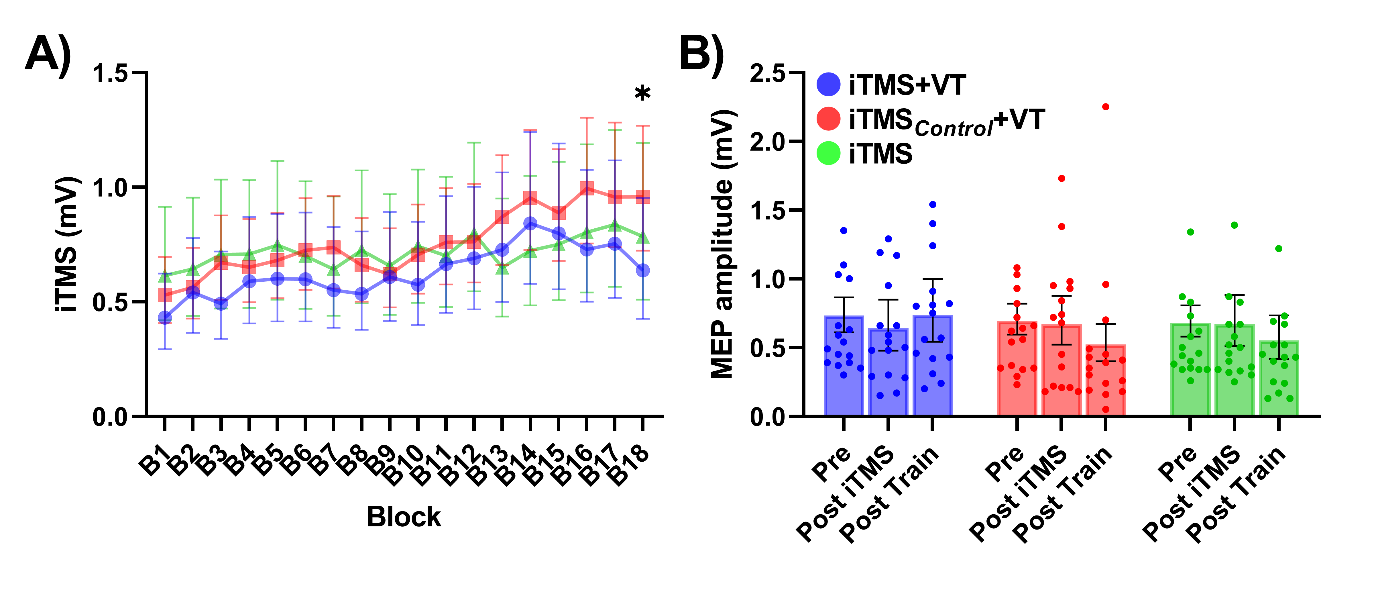


***Figure 2.*** *Corticospinal excitability changes by iTMS and VT. (A) MEP amplitudes during iTMS, averaged over 10 consecutive MEP trials. (B) TS MEP amplitudes before and after iTMS and VT. Median [89% HDI]. Abbreviations; B, block; iTMSsham, control I-wave periodicity repetitive transcranial magnetic stimulation; iTMS, I-wave periodicity repetitive transcranial magnetic stimulation; MEP, motor-evoked potential; VT, visuomotor task.*

*Effects of iTMS on visuomotor training.*

Performance during VT is shown in figure 3. Error (Fig. 3A) was consistently and significantly reduced (all *pd* = 100%, 0% in ROPE) during training (B1-B8) and compared to baseline (Pre). Error was also consistently reduced during B5 (*pd* = 98.1%) and B6 (*pd* = 98.4%) compared to B2, but these were not practically significant (1.4%-2.4% in ROPE). Despite the reduction in error, there was insufficient evidence to accept or reject the null hypothesis for comparisons between sessions (*pd* = 68.5%-82.7%, 12.7%-36.5% in ROPE). MT (Fig. 3B) also consistently reduced throughout VT and compared to baseline (*pd* = 97.2%-100%, 0% in ROPE for significant comparisons). Furthermore, MT was consistently faster during B8 of iTMS+VT compared to iTMS*_Control_*+VT session (*pd* = 95.4%), but this was not practically significant (29.1% in ROPE). Skill also consistently and significantly increased throughout VT and compared to baseline (*pd* = 99.0%-100%, 0% in ROPE for significant comparisons), and skill during B2 and B5 of iTMS+VT was increased compared to iTMS*_Control_*+VT session, with these effects being consistent (*pd* = 98.1-98.4%) and significant (0% in ROPE). In order to account for any potential differences in visuomotor performance at baseline, the analysis of performance measures was repeated using data that were expressed as a percentage of baseline. Using this approach, the effects of session on MT and skill failed to provide sufficient evidence to reject or accept the null hypothesis (*pd* = 76.5%-76.6%, 23.0%-82.7% in ROPE). All other results were consistent with the original analysis of non-normalised data, indicating performance improvement throughout VT.


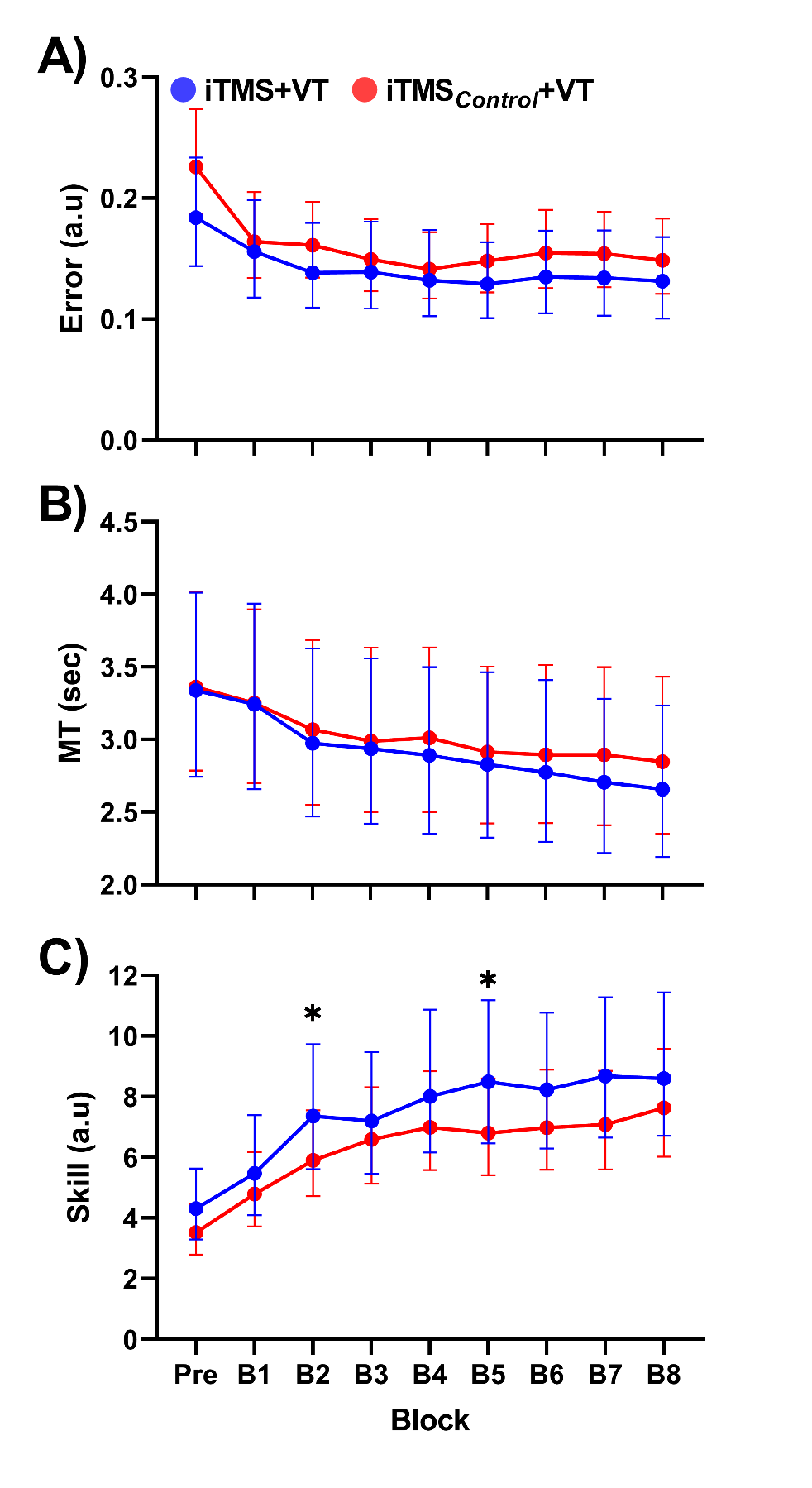


*Figure 3. Changes in motor skills over blocks. Panels (A, B, C) represent error, MT, and skill before and after iTMS, respectively. Median [89% HDI]. Abbreviations; B, block; iTMSsham, control I-wave periodicity repetitive paired-pulse transcranial magnetic stimulation; iTMS, repetitive I-wave periodicity paired-pulse transcranial magnetic stimulation; MT, movement time; VT, visuomotor task.*

**References**

Barr, D. J. (2013). Random effects structure for testing interactions in linear mixed-effects models, Front Psychol. **4:** 328.

Bürkner, P.-C. (2017). "brms: An R package for Bayesian multilevel models using Stan." J Stat Softw **80**: 1-28.

Cash, R., N. Benwell, K. Murray, F. Mastaglia and G. Thickbroom (2009). "Neuromodulation by paired-pulse TMS at an I-wave interval facilitates multiple I-waves." Exp Brain Res **193**(1): 1-7.

Gabry, J., D. Simpson, A. Vehtari, M. Betancourt and A. Gelman (2019). "Visualization in Bayesian workflow." J R Stat Soc Ser A Stat Soc **182**(2): 389-402.

Gelman, A. and D. B. Rubin (1992). "Inference from iterative simulation using multiple sequences." Stat Sci **7**(4): 457-472.

Hand, B. J., A. Merkin, G. M. Opie, U. Ziemann and J. G. Semmler (2023). "Repetitive paired-pulse TMS increases motor cortex excitability and visuomotor skill acquisition in young and older adults." Cereb Cortex: bhad315.

Kidgell, D. J., J. Mason, A. Frazer and A. J. Pearce (2016). "I-wave periodicity transcranial magnetic stimulation (iTMS) on corticospinal excitability. A systematic review of the literature." Neuroscience **322**: 262-272.

Kruschke, J. K. (2018). "Rejecting or accepting parameter values in Bayesian estimation." Adv Methods Pract Psychol Sci **1**(2): 270-280.

Lenth, R. (2023). "emmeans: Estimated Marginal Means, aka Least-Squares Means (R package version 1.8.5)." from <https://CRAN.R-project.org/package=emmeans>.

Liao, W. Y., G. M. Opie, U. Ziemann and J. G. Semmler (2023). "Modulation of dorsal premotor cortex differentially influences I‐wave excitability in primary motor cortex of young and older adults." J Physiol.

Lo, S. and S. Andrews (2015). "To transform or not to transform: using generalized linear mixed models to analyse reaction time data." Frontiers in Psychology **6**.

Makowski, D., M. S. Ben-Shachar, S. A. Chen and D. Lüdecke (2019). "Indices of effect existence and significance in the Bayesian framework." Front Psychol **10**: 2767.

Opie, G. M., J. M. Hughes and R. Puri (2023). "Healthy ageing influences how the shape of alpha and beta oscillations change during reaction time tasks." bioRxiv: 2023.2010.2016.562636.

Puri, R. and M. R. Hinder (2022). "Response bias reveals the role of interhemispheric inhibitory networks in movement preparation and execution." Neuropsychologia **165**: 108120.

Puri, R., R. J. St George and M. R. Hinder (2023). "Investigating the role of contextual cues and interhemispheric inhibitory mechanisms in response-selective stopping: a TMS study." Cogn Affect Behav Neurosci **23**(1): 84-99.

Team, P. (2023a). RStudio: Integrated Development Environment for R., Posit Software, PBC, Boston, MA.

Team, R. C. (2023b). R: A language and environment for statistical computing., R Foundation for Statistical Computing.

Thickbroom, G. W., M. L. Byrnes, D. J. Edwards and F. L. Mastaglia (2006). "Repetitive paired-pulse TMS at I-wave periodicity markedly increases corticospinal excitability: a new technique for modulating synaptic plasticity." Clin Neurophysiol **117**(1): 61-66.
